# Role of complement C1q/C3-CR3 signaling in brain injury after experimental intracerebral hemorrhage and the effect of minocycline treatment

**DOI:** 10.3389/fimmu.2022.919444

**Published:** 2022-09-15

**Authors:** Yonghe Zheng, Linfeng Fan, Siqi Xia, Qiguo Yang, Zhihua Zhang, Huaijun Chen, Hanhai Zeng, Xiongjie Fu, Yucong Peng, Chaoran Xu, Kaibo Yu, Fuyi Liu, Shenglong Cao

**Affiliations:** Department of Neurosurgery, The Second Affiliated Hospital of Zhejiang University School of Medicine, Hangzhou, China

**Keywords:** C1q, CR3, intracerebral hemorrhage, microglia, minocycline

## Abstract

**Aim:**

The complement cascade is activated and may play an important pathophysiologic role in brain injury after experimental intracerebral hemorrhage (ICH). However, the exact mechanism of specific complement components has not been well studied. This study determined the role of complement C1q/C3-CR3 signaling in brain injury after ICH in mice. The effect of minocycline on C1q/C3-CR3 signaling-induced brain damage was also examined.

**Methods:**

There were three parts to the study. First, the natural time course of C1q and CR3 expression was determined within 7 days after ICH. Second, mice had an ICH with CR3 agonists, LA-1 or vehicle. Behavioral score, neuronal cell death, hematoma volume, and oxidative stress response were assessed at 7 days after ICH. Third, the effect of minocycline on C1q/C3-CR3 signaling and brain damage was examined.

**Results:**

There were increased numbers of C1q-positive and CR3-positive cells after ICH. Almost all perihematomal C1q-positive and CR3-positive cells were microglia/macrophages. CR3 agonist LA-1 aggravated neurological dysfunction, neuronal cell death, and oxidative stress response on day 7 after ICH, as well as enhancing the expression of the CD163/HO-1 pathway and accelerating hematoma resolution. Minocycline treatment exerted neuroprotective effects on brain injury following ICH, partly due to the inhibition of C1q/C3-CR3 signaling, and that could be reversed by LA-1.

**Conclusions:**

The complement C1q/C3-CR3 signaling is upregulated after ICH. The activation of C1q/C3-CR3 signaling by LA-1 aggravates brain injury following ICH. The neuroprotection of minocycline, at least partly, is involved with the repression of the C1q/C3-CR3 signaling pathway.

## 1 Introduction

Intracerebral hemorrhage (ICH) is a severe form of stroke caused by the rupture of a blood vessel, which releases blood directly into the brain parenchyma (1). Extravasated blood initiates the coagulation cascade and a hematoma subsequently forms (2, 3). The size of the hematoma is an independent risk factor for early deterioration and long-term prognosis in ICH patients (4). The hematoma has the initial mass effect and subsequent injury response by releasing hemoglobin and degradation products. The hematoma resolution after ICH depends on the processes of hematoma lysis and hematoma clearance (5). Hematolysis after ICH results in the release of hemoglobin and degradation products, contributing to secondary brain injury. Microglia/infiltrating macrophages phagocytize erythrocytes and degradation products that contain potentially neurotoxic and pro-inflammatory components (5). Many studies have found that the complement system plays an important role in hematoma-mediated pathological processes after ICH.

Complement, as a significant part of the innate immune system, contributes to cytolysis and phagocytosis, which are related to hematoma resolution (6). Our previous study indicated that the terminal complex (C5b-9) of the complement cascade, also called the membrane attack complex (MAC), is capable of attaching to and perforating the erythrocyte membrane and further leading to hematoma lysis after ICH (7). Pharmacological and genetic inhibition of complement attenuated hematoma lysis and brain injury after ICH which further confirms the importance of complement components in hematoma lysis (8, 9). Complement-mediated phagocytosis is accomplished by specific recognition of bound complement components using the corresponding complement receptors on the phagocytes. C1q induces the generation of C3 convertase, which cleaves the complement C3 into C3a and C3b. C3b and its degradation product iC3b are deposited on the “tagged” structures and incorporated effectively by phagocytes, which express complement receptor 3 (CR3, also known as CD11b/CD18). The role of C1q/C3-CR3 signaling in microglial phagocytosis has been widely studied in the context of synaptic pruning, the clearance of myelin and amyloid β (Aβ), as well as the removal of several pathogens of the nervous system (10–12). In the process of hematoma clearance, the haptoglobin (Hp)-CD163-heme oxygenase-1(HO-1) is the major pathway of microglia/macrophage-mediated hematoma clearance. However, the role of complement C1q/C3-CR3 signaling in the pathophysiological process after ICH and its interaction with the CD163/HO-1 system remains unclear.

Minocycline, a broad-spectrum tetracycline, presents neuroprotective effects on post-ICH brain injury. The underlying multiple mechanisms include inhibition of microglia activation, the reduction of brain iron deposition, anti-neuroinflammation, and alleviation of blood–brain barrier disruption (13). Our team has demonstrated that minocycline reduces ICH-related perihematomal iron accumulation and brain injury in aged rats with a reliable and noninvasive MRI mapping method (14). Moreover, minocycline is proven to alleviate visceral hypersensitivity by inhibiting complement C1q/C3-CR3 signaling-mediated microglial synaptic remodeling in visceral pain (15). However, the effect of minocycline treatment on C1q/C3-CR3 signaling after ICH has not yet been fully explored.

Based on the evidence above, we determined the role of C1q/C3-CR3 signaling in brain injury and hematoma resolution after ICH. We then examined whether minocycline can attenuate ICH-brain injury by affecting C1q/C3-CR3 signaling.

## 2 Materials and methods

### 2.1 Animal preparation

All animal experiments were approved and supervised by the Institutional Ethics Committee of the Second Affiliated Hospital of Zhejiang University School of Medicine. Adult male C57BL mice (aged 8–10 weeks, weighing 22–25 g) were purchased from the SLAC Laboratory Animal Company Limited (Shanghai, China). The mice were raised in a temperature-controlled (25°C ± 1°C) and humidity-controlled (60 ± 5%) room under a 12-h light/dark cycle. Animals have free access to food and water under specific pathogen-free conditions.

### 2.2 ICH model

The autologous blood injection models of ICH were performed as previously described. Briefly, mice were anesthetized by an intraperitoneal injection of pentobarbital sodium (40 mg/kg, 1%). Autologous blood was acquired from the venous artery. The mice were placed on the stereotaxic frame and drilled a skull hole 2.5 mm right to the bregma at a 5° angle toward the midline. A 26-gauge syringe was inserted 3.5mm ventrally into the right basal ganglia. Approximately 30 μl of whole blood was injected at 3 ul/min by a micro-injection pump. Mice in the sham group received the same treatment, including needle insertion, but without blood injection.

### 2.3 RNA sequencing analysis

Our group previously used RNA sequencing analysis to compare the expression levels of all transcripts between ICH and sham (16). Based on the results of RNA sequencing, we investigated the difference in the transcription of complement components between ICH and sham. Furthermore, we calculated the relative transcriptive level of complement-related genes between ICH and sham *via* ssGESA. The accession number of the RNA sequence data is GSE206971 (GEO).

### 2.4 Clinical data collection

We included 60 ICH patients admitted to the Second Affiliated Hospital of Zhejiang University (Hangzhou, China) from January 2018 to January 2022 in our retrospective observational study, in which ICH patients were divided into a <30 ml group (n = 30) and a ≥30 ml group (n = 30) based on hematoma volume. Sixty healthy people on physical examination during the same period were included as a sham group. The inclusion criteria of ICH patients were as follows: Patients confirmed with ICH in basal ganglia based on CT, patients admitted to the hospital within 24 h after the onset of ICH, patients who had not recently used any immune preparations or anti-inflammatory drugs. The exclusion criteria from the study were as follows: Patients with ICH in other brain areas, patients accompanied by infection, patients with other organ inflammation, patients comorbid with a malignant cancer, cardio-pulmonary failure, or hepatic or kidney function obstacle, and patients suffering from traumatic intracerebral hemorrhage or subarachnoid hemorrhage. In general clinic data, the age of the sham group was 61.1 ± 8.97 (ranging from 50 to 95), with 13 females and 47 males. The age of the ICH group was 63.15 ± 9.84 (ranging from 50 to 89), with 14 females and 46 males. There is no significant difference between age and sex (P >0.05). The levels of complement C1q and C3 in the serum of ICH patients and healthy people were collected. The informed consent in our study (the ethic number: I2019001510) was approved by the Ethics Committee of the Second Affiliated Hospital of Zhejiang University.

### 2.5 Study design

There are three independent parts in this study, and each experiment is repeated three times to obtain reliable data. The experimental group is shown in **Supplementary Figure 1**, and the experimental design is shown in **Supplementary Figure 2**.

#### 2.5.1 Experiment 1

Mice were randomly divided into the following four groups to investigate the expression pattern and distribution of C1q and CR3 expression after ICH: sham, ICH1d, ICH3d, ICH7d, and ICH14d. Immunofluorescence staining for the time course of C1q and CR3 expression was performed in the sham, ICH1d, ICH3d, ICH7d, and ICH14d groups. Additionally, cellular locations of C1q and CR3 in the samples from ICH were analyzed by double immunofluorescence staining.

#### 2.5.2 Experiment 2

Mice were randomly assigned into the following three groups to study the role of CR3 after ICH: sham, ICH+ vehicle1, and ICH+ Leukadherin-1(LA-1) groups. The ICH+ vehicle1 group was treated with the vehicle used for LA-1 after ICH. The ICH+ LA-1 group was treated with LA-1 after ICH for 7 days. The neurological scores were estimated in each group at ICH1, 3, 7, and 14 days after ICH. At 7 days after ICH conduction, Fluoro-Jade C (FJC) staining, DHE staining, immunofluorescence, and Western blotting were performed. To evaluate hematoma resolution, hematoma volume was measured in the ICH+ vehicle1 and ICH+ LA-1 groups.

#### 2.5.3 Experiment 3

The effects of minocycline on C1q/C3-CR3 signaling and brain damage were investigated. Mice were randomly divided into the following three groups: sham, ICH+ vehicle2, and ICH+ Minocycline (Mino) groups. The ICH+ vehicle2 group was treated with the vehicle used for Mino after ICH. The ICH+ Mino group was treated with minocycline after ICH for 7 days. The neurological scores were evaluated in each group at ICH1, 3, 7, and 14 days after ICH. Fluoro-Jade C staining, DHE staining, immunofluorescence, and Western blotting were performed 7 days after ICH conduction. Furthermore, hematoma volume was evaluated in the ICH+ vehicle2 and ICH+ Mino groups. Additionally, mice were randomly assigned to the ICH+ vehicle3, ICH+ Mino+ vehicle1, and ICH+ Mino+ LA-1 groups. The ICH+ vehicle3 group was treated with the vehicle used for LA-1 and the vehicle used for Mino after ICH. The ICH+ Mino+ vehicle1 group was treated with Mino and the vehicle used for LA-1 after ICH for 7 days. The ICH+ Mino+ LA-1 group was treated with Mino and LA-1 after ICH for 7 days. The neurological scores were estimated in each group at ICH1, 3, 7, and 14 days after ICH. Immunofluorescence and FJC staining were performed 7 days after ICH conduction.

### 2.6 Drug administration

Leukadherin-1 (S8306, Selleck, China) was dissolved in 2% DMSO as described previously (17). The minocycline (from Maokang Biotechnology Co., Ltd., Shanghai) was dissolved in PBS. The ICH+ Leukadherin-1 (ICH+ LA-1) group received Leukadherin-1 2 mg/kg intraperitoneally after ICH followed by administration once daily for 7 days. The ICH+ vehicle1 group was injected with an equal volume of 2% DMSO for 7 days. The ICH+ Minocycline (ICH+ Mino) group is treated with minocycline (50 mg/kg) *via* oral gavage after ICH, followed by daily gavage until sacrifice. The ICH+ vehicle2 group was administered the same volume of PBS for 7 days. The ICH+ Minocycline+ Leukadherin-1(ICH+Mino+LA-1) group was administered with 2 mg/kg Leukadherin-1 intraperitoneally and 50 mg/kg minocycline orally after ICH, followed by administrations once daily for 7 days. The ICH+ Mino+ vehicle1 group was given 50 mg/kg minocycline orally and an equal volume of 2% DMSO followed by administration once daily for 7 days. The ICH+ vehicle3 group was given an equal volume of 2% DMSO and PBS for 7 days.

### 2.7 Behavioral tests

Neurobehavioral functions were estimated by three behavioral tests, including forelimb placing test, cylinder test, and corner turn test according to previous studies (18).

### 2.8 Immunofluorescence staining

Immunofluorescence staining was conducted as described previously (19). Coronal sections were washed with PBS and incubated with 5% BSA and 0.3% Triton X-100 for 1 h at room temperature. The brain slices were then incubated with primary antibodies overnight at 4 °C. The primary antibodies in our study were as follows: goat anti-Iba-1 antibody (1:500, ab5076, Abcam), mouse anti-NeuN antibody (1:500, ab104224, Abcam), mouse anti-GFAP antibody (1:500, ab10062, Abcam), rat anti-CR3/CD11b antibody antibody (1:300, ab8878, Abcam), mouse anti-C1q antibody (1:300, ab71940, Abcam), and rabbit anti-CD163 antibody (1:200, ab182422, Abcam). The brain slices were incubated with secondary antibodies at room temperature for 2 h. Finally, three random fields of vision were examined in each slice to acquire the average number of positive cells using a fluorescence microscope (Leica, Mannheim, Germany).

### 2.9 FJC staining

Neuronal damage was evaluated using FJC staining (Fluoro-Jade C Ready-to-Dilute Staining Kit, Biosensis, CA, USA) at 7 days after ICH according to the instructions of the manufacturer. Under ×200 magnification, FJC-positive cells were counted within the ipsilateral cerebral cortex and averaged from three random fields using a fluorescence microscope (Leica, Mannheim, Germany).

### 2.10 Detection of reactive oxygen species

To detect the level of ROS and cell types generating ROS after ICH, freshly prepared frozen brain sections (10 μm) were double-immunostained with dihydroethidium (DHE, 2 mM, Beyotime, China) and immunofluorescence. The primary antibodies in this experiment were as follows: anti-iba-1 antibody (1:500, ab10062, Abcam), anti-NeuN antibody (1:500, ab104224, Abcam), and anti-GFAP antibody (1:500, ab10062, Abcam). Images were examined using a fluorescence microscope (Leica, Mannheim, Germany) and evaluated with ImageJ software.

### 2.11 Western blotting

Western blotting was conducted as described previously (16). Briefly, perfusion of the brain tissue was conducted using 0.1 mM PBS after euthanasia, and basal ganglia was sampled. Proteins from the brain samples were homogenized using RIPA lysis buffer. The same amounts of protein (50 μg/10 μl) were loaded onto sodium dodecyl sulfate-polyacrylamide gels (SDS-PAGE). The proteins were electrophoresed and transferred to PVDF membranes. The membranes were blocked with non-fat dry milk buffer for 1 h and incubated with the primary antibodies at 4 °C overnight. The primary antibodies used in this study were as follows: rabbit anti-heme oxygenase 1 antibody (1:5,000, ab68477, Abcam) and mouse anti-beta actin antibody (1:10,000, 66009-1-lg, Proteintech). The membranes were then incubated with the horseradish-peroxidase-conjugated secondary antibody for 1 h at room temperature. Protein bands were visualized using the Immobilon ECL Ultra Western HRP Substrate reagent kit (EMD Millipore Corporation, Billerica, MA, USA), and proteins were quantified by ImageJ software.

### 2.12 Statistical analysis

Continuous data were presented asmean ± standard deviation (SD) or median (interquartile range) based on the normality and homogeneity of variance. For the data satisfying normal distribution and homogeneity of variance, two-way analysis of variance (ANOVA) followed by a Tukey’s multiple comparison test was used to evaluate significant differences among three or more groups, and an unpaired t-test was applied to compare differences between two groups. For the data with unequal variation, Brown-Forsythe and Welch’s ANOVA were used to evaluate significant differences among three or more groups, and Welch’s t-test was introduced to make explicit the differences between two groups. For the data with abnormal distribution, the Kruskal–Wallis test was used to show the differences among three or more groups, and the Mann–Whitney U test was introduced to explicitly show the differences between two groups. For comparisons of time-series data in different groups, two-way ANOVA was used. P <0.05 was considered statistically significant. GraphPad Prism 8.0 and SPSS software 23.0 were used for statistical analyses.

## 3 Results

### 3.1 The complement system is activated, and the expression of C1q and CR3 is upregulated after ICH

**Figure 1:**
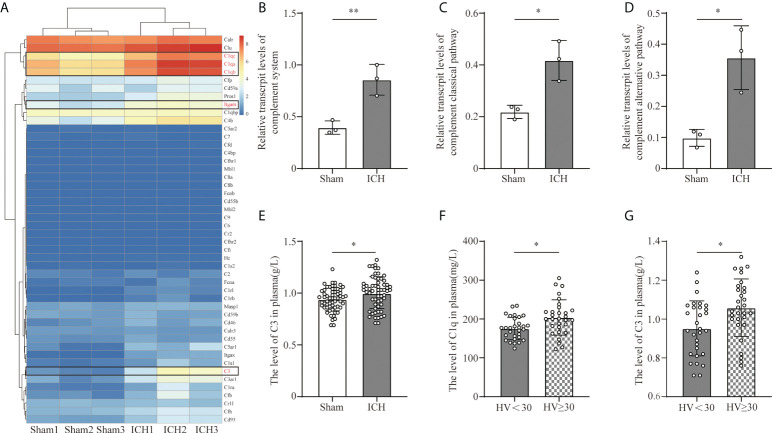
Complement activation after ICH. **(A)** Heat map of complement genes in mouse peri-hematomal brain tissue 3 days after ICH compared with the levels in the sham group. C1qa, C1qb, C1qc (C1q gene), C3 (C3 gene), and Itgam (CR3 gene) were significantly upregulated. n = 3. **(B)** Quantitative analysis of the complement system in transcriptive level in the hematoma edge after ICH. n = 3. **P <0.01 by two-tail Student’s t-test. **(C)** Quantitative analysis of the complement classic pathway at the transcriptive level in hematoma edge after ICH. n = 3. *P <0.05 by two-tail Student’s t-test. **(D)** Quantitative analysis of the complement alternative pathway in transcriptive level in the hematoma edge after ICH. n = 3. *P <0.05 by two-tail Student’s t-test. **(E)** Quantification of serum C3 in healthy people and ICH patients. n = 60. *P <0.05 by Welch’s t-test. **(F)** Quantification of C1q in plasm in patients with hematoma volume <30 ml and hematoma volume ≥30 ml. n = 30. *P <0.05 by Welch’s t-test. **(G)** Quantification of C3 in plasm in patients with hematoma volume <30 ml and hematoma volume ≥30 ml. n = 30. *P <0.05 by two-tail Student’s t-test. Data are represented as mean ± SD.

To investigate the transcriptive levels of complement genes after ICH, we analyzed the mRNA expression profile of complement in the mouse brain by RNA seq. We found that the main complement component genes were upregulated after ICH, among which C1qa, C1qb, C1qc (C1q gene), C3(C3 gene), and Itgam (CR3 gene) were obviously increased (**Figure 1A**). We calculated the relative transcriptive levels and revealed that gene expressions in the complement system and the classical pathway, as well as complement alternative pathway, prominently increased after ICH compared to the sham group *via* ssGESA (**Figures 1B–D**). However, complementation of the lectin pathway had no difference between the ICH and sham groups (**Supplementary Figure S3**).

C1q and C3 are the initiating factors of complement cascade activation. To investigate the effect of ICH on the levels of C3 and C1q, we collected the serum C3 and C1q levels from healthy people and ICH patients included in our retrospective observational study. The level of C3 was higher in ICH patients than in healthy people (**Figure 1E**). Moreover, the levels of C3 and C1q were higher in patients with larger hematoma volumes (**Figures 1F, G**).

**Figure 2:**
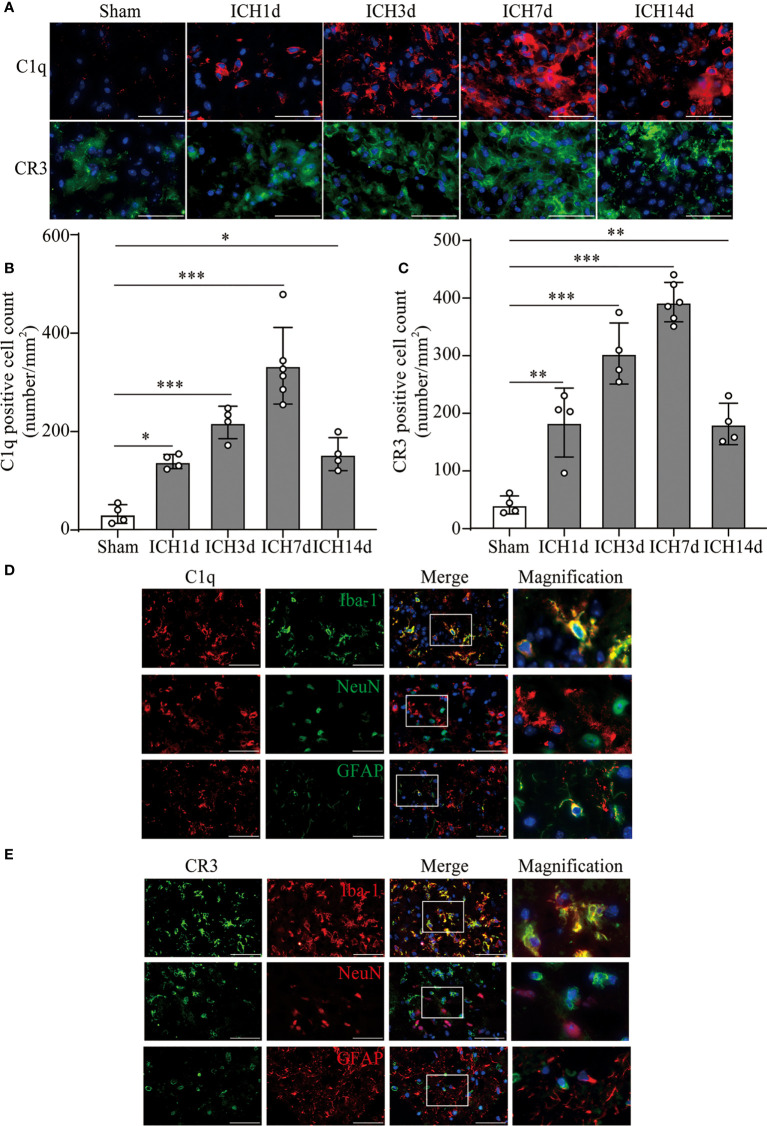
The activation of C1q and CR3 after ICH. **(A–C)** Representative immunofluorescent image and quantitative analysis of C1q and CR3 after ICH. n = 4 in the Sham, ICH1d, ICH3d, and ICH14d groups and n = 6 in the ICH7d group. *P <0.05, **P <0.01, ***P <0.001 by one-way ANOVA. (**D, E**) Representative microphotographs of immunofluorescence double staining showing the localization of both CR3 and C1q with Iba-1 (microglia marker), NeuN (neuronal marker), and GFAP (astrocyte marker) after ICH3d. n = 3. Scale bar = 50 μm. Data are represented as mean ± SD.

Based on the results of RNA sequence and clinic data, we further investigated the levels of C1q and CR3 expression in the perihematomal area at different time points in the ICH model. The expression of C1q and CR3 was significantly increased around the hematoma, whereas there were almost no positive cells in the sham group. C1q and CR3-positive cells gradually increased with marked accumulation on day 7 after ICH (**Figures 2A–C**). Co-staining showed that both C1q and CR3 were mainly expressed on Iba-1-positive microglia/macrophage around hematoma after ICH3d (**Figures 2D, E**).

### 3.2 CR3 agonist LA-1 exacerbates neurological dysfunction, neuronal cell death, and oxidative stress response

**Figure 3:**
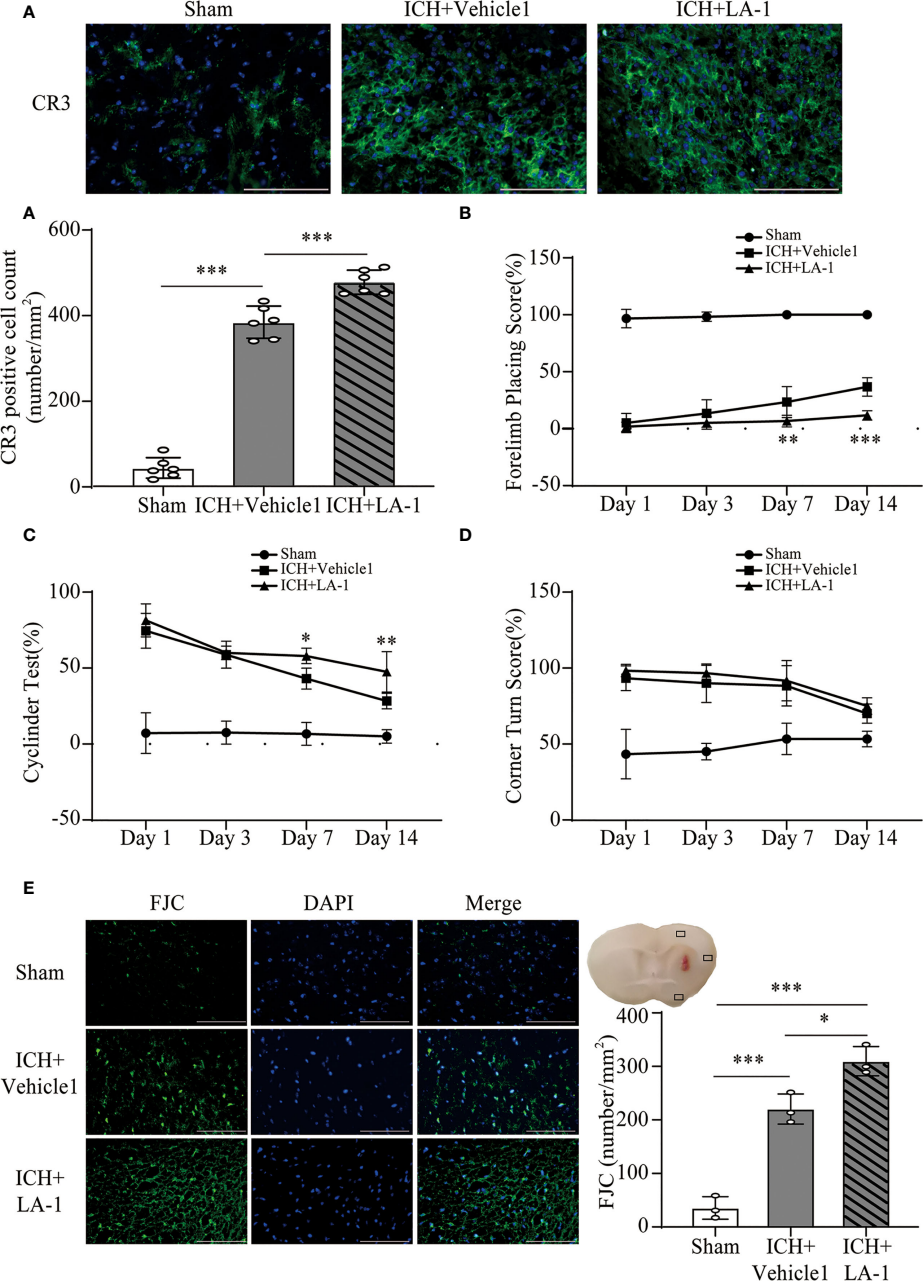
The effect of CR3 activation on brain damage after ICH. **(A)** Representative immunofluorescence images and quantitative analysis of CR3 positive cell. n = 6. ***P <0.001 by one-way ANOVA. **(B–D)** Quantification of neurological function in the forelimb placing test **(B)**, cylinder test **(C)**, and corner turn test **(D)** at days 1, 3, 7, and 14. n = 6. *P <0.05, **P <0.01 versus ICH+ vehicle1 group by two-way ANOVA. **(E)** Representative immunofluorescence images and quantitative analysis of FJC-positive cells in the ipsilateral cerebral cortex. n = 3. *P <0.05, **P <0.01, ***P <0.001 by one-way ANOVA. Scale bar = 100 μm. Data are represented as mean ± SD.

We introduced Leukadherin-1 (LA-1), a CR3 agonist, to elucidate the role of CR3 in ICH-induced brain injury. The number of CR3-positive cells in the perihematomal area increased significantly in the ICH+ LA-1 group compared with the ICH+ vehicle1 group (**Figure 3A**). Then, three different behavior tests were used to assess the effect of LA-1 on neurological deficits according to previous studies (18). Compared with the sham operation group, neurological impairment occurred on days 1, 3, and 7, 14 after ICH in these behavioral tests. The LA-1 intervention significantly aggravated neurological dysfunction in the forelimb placing test and cylinder test on days 7 and 14 after ICH (**Figures 3B, C**). However, there was no statistical difference between the ICH+ LA-1 group and the ICH+ vehicle1 group in the corner turn test (**Figure 3D**).

**Figure 4:**
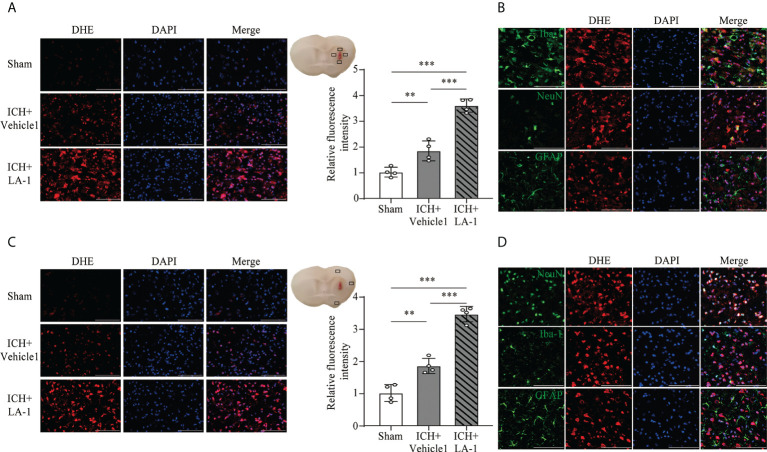
The effect of CR3 activation on oxidative stress after ICH**. (A, C)** Representative microphotographs and quantitative analysis of relative fluorescence intensity of DHE in perihematomal area and ipsilateral cerebral cortex. n = 4. **P <0.01, ***P <0.001 by one-way ANOVA. **(B)** Representative microphotographs of immunofluorescence double staining showing the localization of DHE with Iba-1 (microglia marker), NeuN (neuronal marker), and GFAP (astrocyte marker) in the perihematomal area after ICH. n = 4. **(D)** Representative microphotographs of immunofluorescence double staining showing the localization of DHE with Iba-1 (microglia marker), NeuN (neuronal marker), and GFAP (astrocyte marker) in the ipsilateral cerebral cortex after ICH7d. n = 4. Scale bar = 100 μm. Data are represented as mean ± SD.

FJC staining was performed to evaluate neuronal cell death after ICH. The results showed that LA-1 significantly increased the number of FJC-positive cells in the ipsilateral cerebral cortex in the ICH+LA-1 group compared with that in the ICH+ vehicle1 group (**Figure 3E**). An oxidative stress response is induced by the accumulation of reactive oxygen species (ROS) and plays an important role in neuronal cell death following ICH (20). Dihydroethidium (DHE) as a fluorescent probe was used for detecting ROS generation. ROS levels were significantly higher in the ICH group than in the sham group, and activation of CR3 by LA-1 markedly enhanced the production of ROS in the perihematomal area and the ipsilateral cerebral cortex (**Figures 4A, C**). Activated phagocytes and nonphagocytic cells are the main sources of ROS following ICH (20). Co-staining immunofluorescence confirmed that ROS was mainly derived from microglia in the hematoma edge and neurons in the ipsilateral cerebral cortex (**Figures 4B, D**).

### 3.3 CR3 agonist LA-1 upregulates CD163/HO-1 expression and hematoma resolution

**Figure 5:**
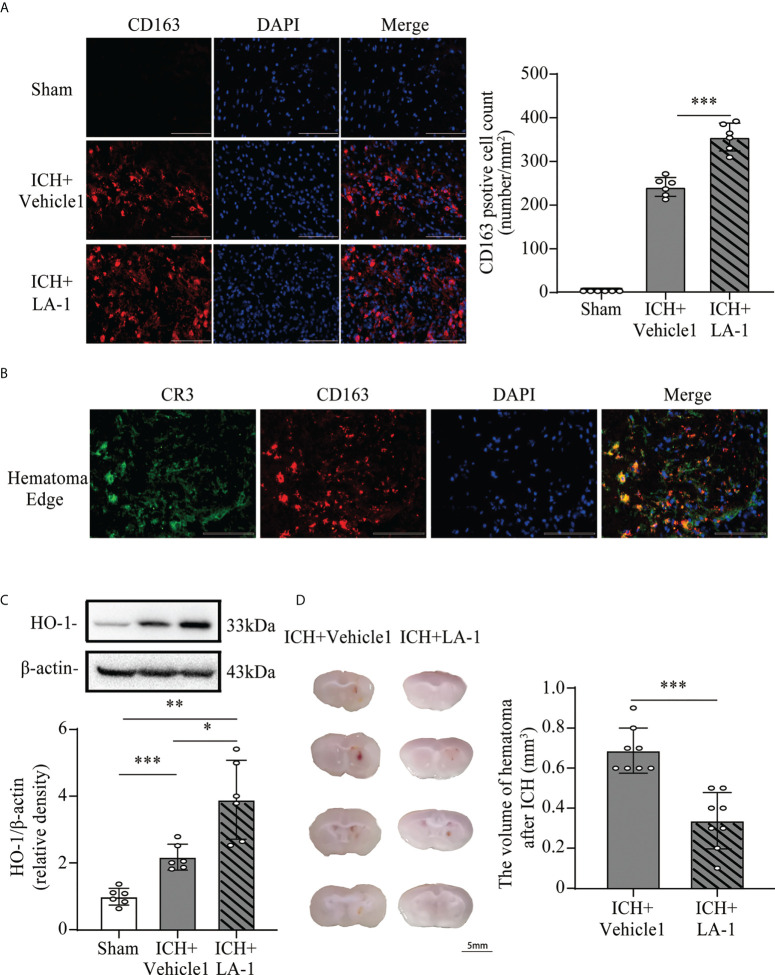
The effect of CR3 activation on the CD163/HO-1 pathway and hematoma resolution after ICH. **(A)** Representative immunofluorescent image and quantitative analysis of CD163 positive cell. Scale bar = 100 μm. n = 6. ***P <0.001 by one-way ANOVA. **(B)** Representative microphotographs of immunofluorescence double staining showing the localization of CR3 with CD163 in the perihematomal area after ICH. Scale bar = 100 μm. n = 3. **(C)** Representative western blotting images and quantitative analysis of HO-1. n = 6. *P <0.05, **P <0.01, ***P <0.001 by Brown-Forsythe and Welch’s ANOVA. **(D)** Representative brain section and quantitative analysis of hematoma volume. n = 8. ***P <0.001 by the Mann–Whitney test. Data are represented as mean ± SD.

Previous studies have indicated that C1q/C3-CR3 signaling is involved in phagocyte-mediated phagocytosis (11, 12, 21). CD163 acts as a main hemoglobin receptor, mediating the intracellular uptake of hemoglobin into microglia/macrophages, and the CD163/HO-1 system is the main pathway for hematoma clearance after ICH (22, 23). To explore the role of pharmacological CR3 activation in microglia-related phagocytosis, the CD163/HO-1 pathway activation and hematoma volume were assessed. CD163-positive cells were located mostly in the perihematomal area, and numbers increased significantly in the ICH+ LA-1 group compared with ICH+vehicle1 group (**Figure 5A**). Double immunofluorescence staining showed that CD163-positive-stained cells were colocalized with CR3-positive cells in the perihematomal area (**Figure 5B**). HO-1 protein levels were upregulated after ICH, and the CR3 agonist LA-1 further upregulated the expression of HO-1 protein in basal ganglia compared with the vehicle-treated ICH group (**Figure 5C**). On day 7 after ICH, the residual hematoma volume was smaller in the ICH+ LA-1 group than in the ICH+ vehicle1 group (**Figure 5D**). The above results suggest that the CR3 agonist LA-1 had effects on upregulating the CD163/HO-1 pathway and promoting hematoma resolution.

### 3.4 Minocycline presents neuroprotection on brain injury after ICH

**Figure 6:**
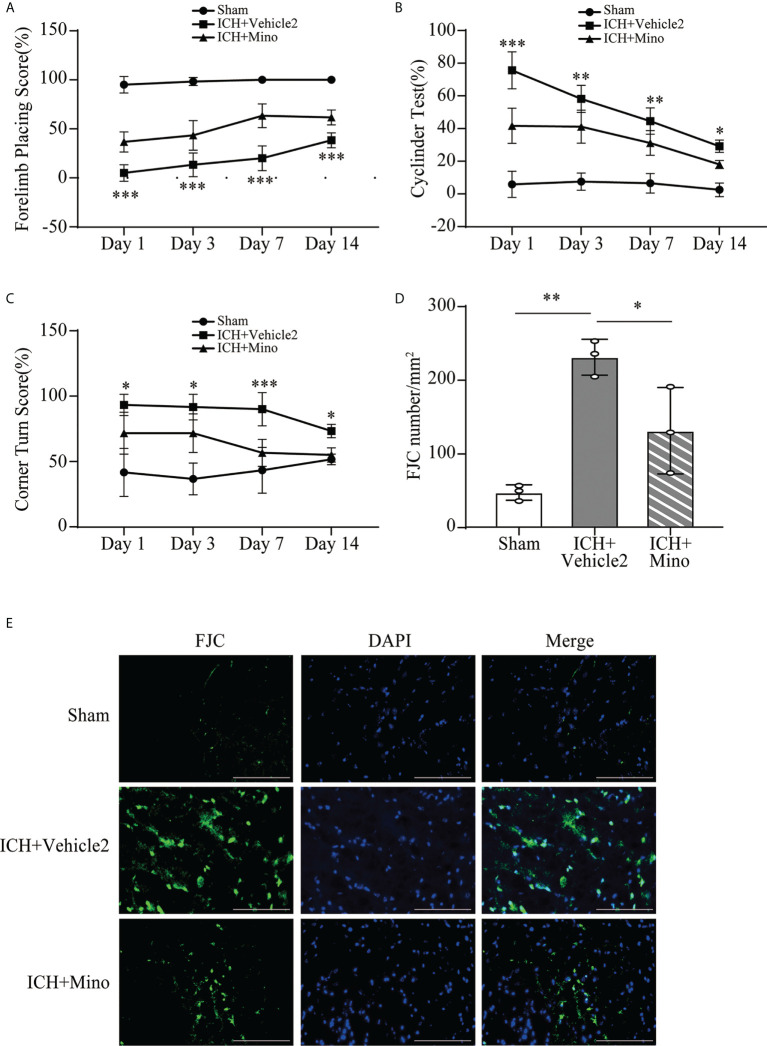
The neuroprotection of minocycline on brain injury after ICH. **(A–C)** Quantification of neurological function in the forelimb placing test **(A)**, cylinder test **(B)**, and corner turn test **(C)** at days 1, 3, 7, and 14. n = 6, *P <0.05, **P <0.01, ***P <0.001 versus the ICH + Mino group by two-way ANOVA. **(D, E)** Representative immunofluorescence images and quantitative analysis of FJC-positive cells in the ipsilateral cerebral cortex. n = 3. *P <0.05, **P <0.01 by one-way ANOVA. Scale bar = 100 μm. Data are represented as mean ± SD.

**Figure 7:**
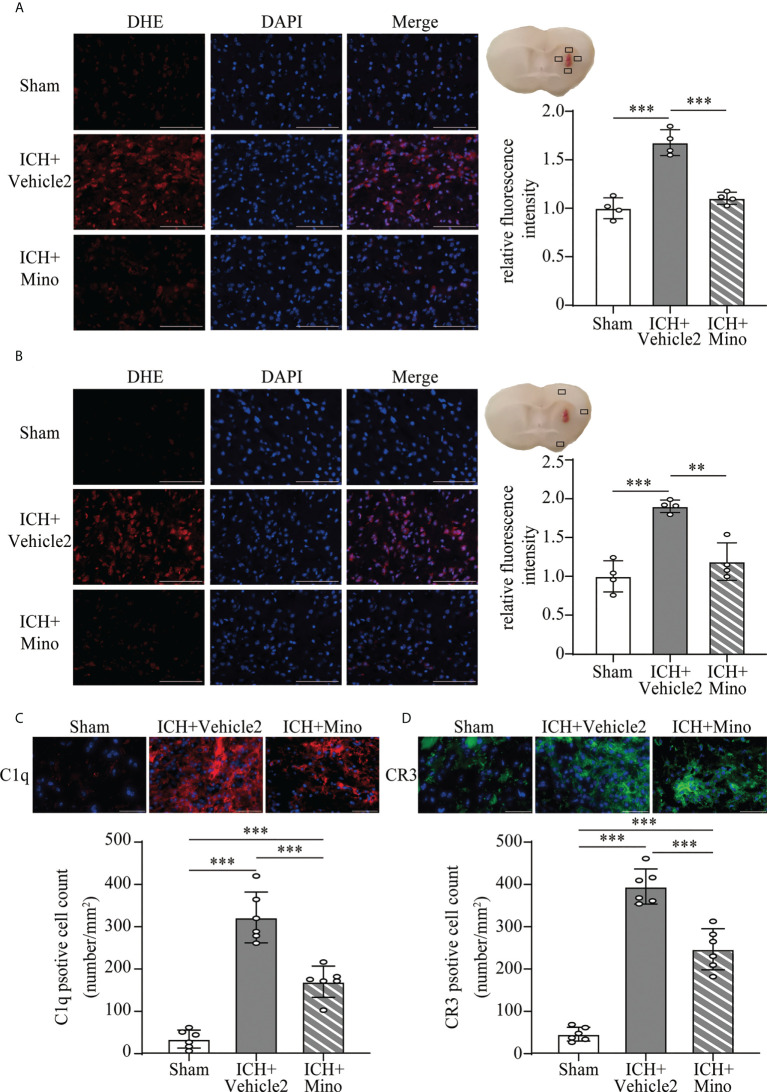
The effect of minocycline on oxidative stress and C1q, CR3 expression after ICH. **(A, B)** Representative microphotographs and quantitative analysis of the relative fluorescence intensity of DHE in the perihematomal area and ipsilateral cerebral cortex after ICH. Scale bar = 100 μm. n = 4. **P <0.01, ***P <0.001 by one-way ANOVA. **(C, D)** Representative immunofluorescent image and quantitative analysis of C1q and CR3 positive cells after ICH. Scale bar = 50 μm. n = 6. ***P <0.001 by way of one-way ANOVA. Data are represented as mean ± SD.

The neuroprotective effects of minocycline on brain injury following ICH *via* intraperitoneal injection have been confirmed by many studies (14, 24, 25). This study was designed to evaluate the effect of minocycline *via* oral gavage after ICH in mice. Oral minocycline treatment improved neurological function in all behavior tests at 1,3,7, and 14 days after ICH, compared with the ICH+ vehicle2 group (**Figures 6A–C**). Consistently, oral minocycline treatment resulted in a significant decrease in the number of FJC-positive cells compared to that in the ICH+ vehicle2 group (**Figures 6D, E**). Moreover, the DHE staining demonstrated that minocycline treatment obviously decreased the level of ROS in the hematoma edge and ipsilateral cortex on day 7 after ICH (**Figures 7A, B**).

### 3.5 Minocycline downregulates C1q/C3-CR3 signaling, reduces CD163/HO-1 expression and hematoma resolution

**Figure 8:**
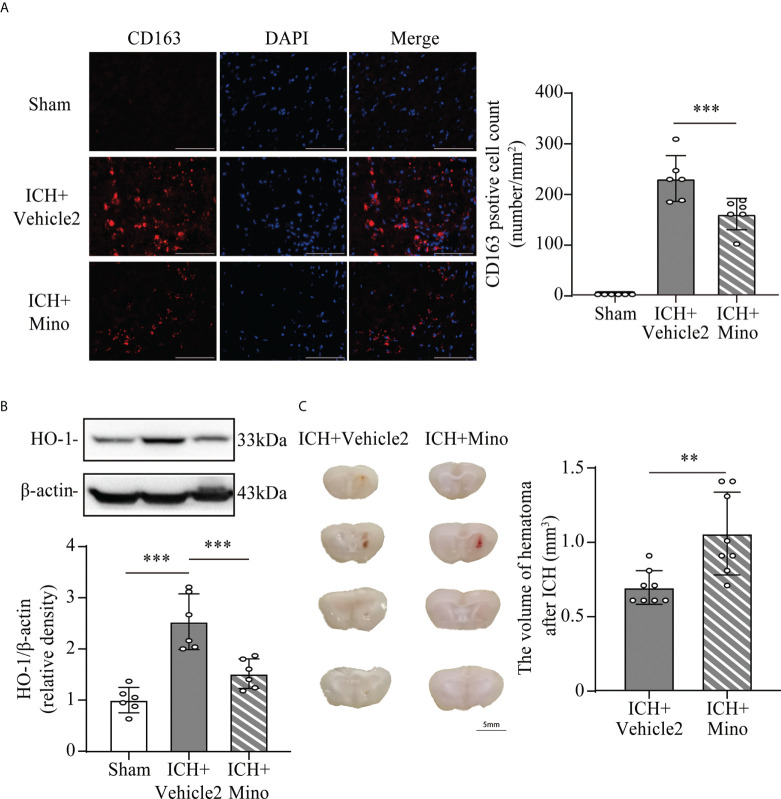
The effect of minocycline on the CD163/HO-1 pathway and hematoma resolution after ICH. **(A)** Representative immunofluorescent image and quantitative analysis of CD163 positive cell. ***P <0.001 by way of one-way ANOVA. Scale bar = 100 μm. n = 6. **(B)** Representative western blotting images and quantitative analysis of HO-1. n = 6. ***P <0.001 by Brown-Forsythe and Welch’s ANOVA. **(C)** Representative brain section and quantitative analysis of hematoma volume. n = 8. **P <0.01 by Mann–Whitney test. Data are represented as mean ± SD.

Based on the fact that the activation of CR3 exacerbates ICH-induced brain injury, we investigated whether minocycline treatment had effects on C1q/C3-CR3 signaling after ICH. Our data demonstrated that the number of C1q and CR3-positive cells was less in the ICH+ Mino group compared to the ICH+ vehicle2 group on day 7 after ICH (**Figures 7C, D**). CR3 activation upregulated CD163/HO-1 expression and promoted hematoma resolution, and these items were also measured in this part of the experiment. Oral minocycline treatment decreased CD163-positive cells in the perihematomal area and reduced HO-1 protein expression in the basal ganglia on day 7 after ICH (**Figures 8A, B**). Minocycline treatment resulted in bigger residual hematoma volume compared to ICH+ vehicle2 group on day 7 after ICH (**Figure 8C**). These data indicated that minocycline downregulated CD163/HO-1 expression and slowed down hematoma resolution, which might be associated with the inhibition of CR3 activation after ICH.

### 3.6 The neuroprotective effects of minocycline are reversed by LA-1

**Figure 9:**
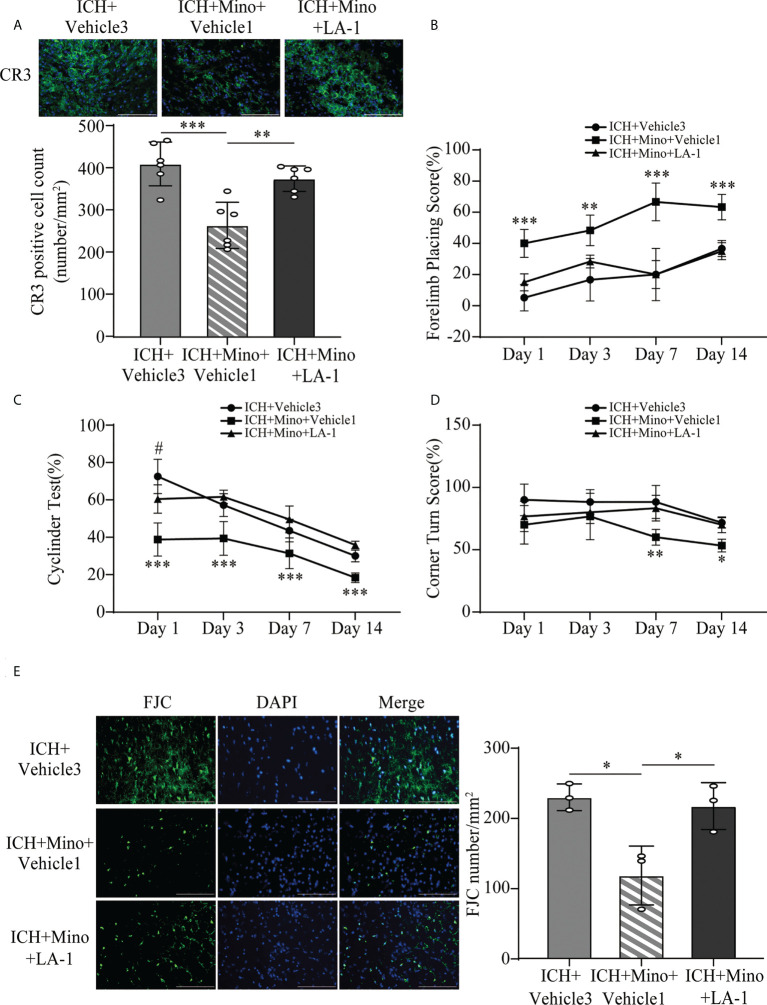
CR3 activation reverses minocycline-mediated neuroprotection against ICH. **(A)** Representative immunofluorescence images and quantitative analysis of CR3 positive cells. n = 6. **P <0.01, ***P <0.001 by one-way ANOVA. **(B–D)** Quantification of neurological function in the forelimb placing test **(B)**, cylinder test **(C)**, and corner turn test **(D)** at days 1, 3, 7, and 14. N = 6. **P <0.01 versus ICH + Mino + LA-1 group, ***P <0.001, ^#^P <0.05 versus ICH + Mino + LA-1 group by two-way ANOVA. **(E)** Representative immunofluorescence images and quantitative analysis of FJC-positive cells in the ipsilateral cerebral cortex. n = 3. *P <0.05 by one-way ANOVA. Scale bar = 100 μm. Data are represented as mean ± SD.

The coincident changes in CR3 expression and post-ICH brain injury induced by oral minocycline treatment encouraged us to investigate whether a CR3 agonist can reverse the minocycline-mediated neurological protection under ICH conditions. The number of CR3-positive cells in the ICH+ Mino group was remarkably reduced while the CR3 agonist LA-1 reversed the minocycline-mediated CR3 inhibition (**Figure 9A**). Behavioral tests were used to examine the role of CR3 in minocycline-mediated behavioral recovery on days 1, 3, 7, and 14 after ICH. Minocycline prominently improved neurological function, whereas the protective effects of minocycline treatment were reserved for CR3 activation (**Figures 9B–D**). FJC staining was used to evaluate neuronal degradation on day 7 after ICH. Minocycline alleviated ICH-induced neuronal damage on day 7 after ICH, and LA-1 aggravated neuronal death after minocycline treatment, almost similar to that in the ICH group (**Figure 9E**). Together, the potentiation of CR3 with LA-1 reversed minocycline-mediated neurological protection.

## 4 Discussion

**Figure 10:**
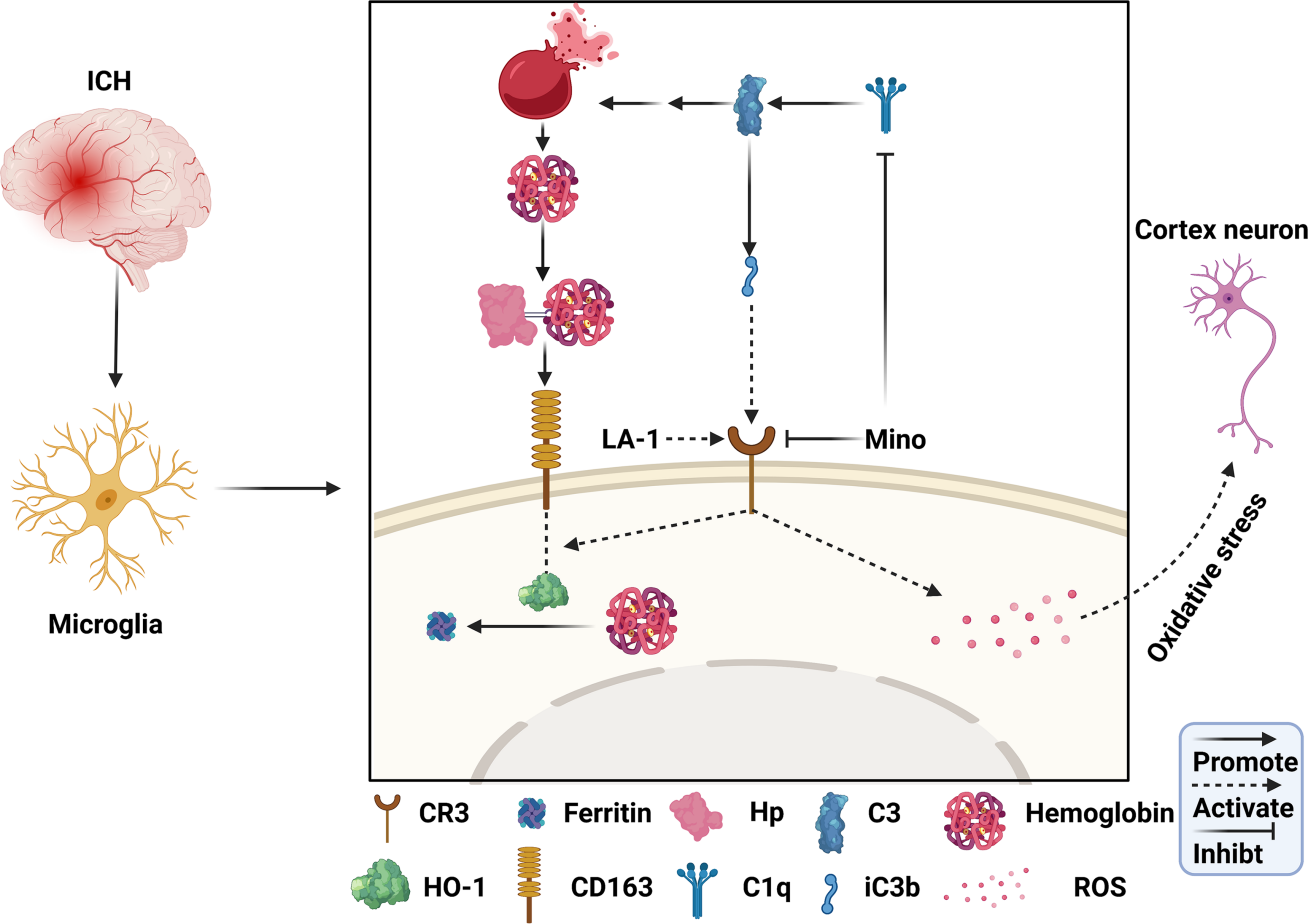
Schematic mechanism of CR3 regulates brain damage and hematoma resolution after ICH and the effect of minocycline treatment. The ICH insult activates the complement cascade. CR3 activation can exacerbate brain injury after ICH by promoting oxidative stress in perihematomal microglia and cortical neurons. Complement-mediated erythrolysis results in the release of hemoglobin, and CR3 activation upregulates the CD163-OH-1 pathway to accelerate hematoma clearance. Minocycline inhibits C1q and CR3, and alleviates brain damage *via* repressing CR3-induced oxidative stress and hematoma resolution.

In this study, we identified the role of C1q/C3-CR3 in the pathological process following ICH and assessed the effect of minocycline on brain injury. The major findings of this study are as follows (**Figure 10**): (1) C1q/C3-CR3 signaling was active with time after ICH; (2) activation of CR3 exacerbated ICH-induced brain injury; (3) CR3 activation upregulated CD163/HO-1 expression and promoted hematoma resolution after ICH; (4) oral minocycline treatment had neuroprotective effects on brain injury after ICH partly by downregulating C1q and CR3; and (5) the effect of oral minocycline on post-ICH brain injury can be reversed by potentiating CR3.

Previous evidence suggested that the complement system was activated after ICH. Current studies have revealed that there was a sixfold increase in C9 around the hematoma 24 h after ICH, and MAC and clusterin also increased in the perihematomal tissue 3 days after ICH (7, 26).The anaphylatoxins C3a and C5a were upregulated after ICH: the level of C3a increased within 5 days after ICH, whereas the level of C5a peaked on day 3 and began to decrease (27). In a clinical study, patients with cerebral hemorrhage had higher C3 levels and lower C4 levels in serum than healthy people (28). Our study confirmed that the complement system and two complement pathways were upregulated after ICH at the transcriptive level in mouse brain tissue. C1q represented the initiating step of the classical complement pathway. The C3 cleavage was the core of the complement cascade and MAC formation. CR3 is the receptor of complement iC3b, the main downstream product from the cleavage of C3 by C1q in the classical complement pathway. We explored C1q, C3, and CR3 expression levels after ICH. We found that C1q and CR3 increased immediately and peaked on day 7 in the brain tissue of ICH mice. Moreover, we examined serum C1q and C3 in ICH patients and healthy people. The results revealed serum C3 was increased in ICH patients, and the levels of serum C1q and C3 serum were positively correlated with hematoma volume. A recent study by Zheng et al. pointed out that patients with neurological function score NIHSS ≥4 have higher serum C3 levels than those with NIHSS <4 after cerebral hemorrhage (28). These results demonstrated that the complement cascade was potentiated after ICH and that the extent of complement activation was related to the severity of ICH-induced brain injury.

Complement contributes to brain edema and brain injury after ICH in multiple ways. The membrane attack complex, the end product of the complement cascade, exacerbates perihematomal neuronal loss *via* erythrolysis (7, 29). The anaphylatoxins C3a and C5a cause chemotaxis of neutrophils and mast cells, which can release oxygen radicals and lysosomal proteases (30, 31). Moreover, astrocytes upregulate and release C3, which binds to C3aR in microglia, promoting neuroinflammation and hydrocephalus after germinal matrix hemorrhage (32). However, few studies have explored the role of other complement components in the pathological process after ICH. Our animal experiments and clinical data showed that C1q/C3-CR3 signaling was significantly upregulated after ICH, and we further explored the role of CR3 activation in ICH-induced brain injury. We observed that LA-1, a CR3 agonist, increased ROS generation, promoted neuronal cell death and aggravated neurological deficits after ICH. Meanwhile, LA-1 upregulated CD163/HO-1 expression and reduced residual hematoma volume after ICH. All these results indicate that when promoting hematoma clearance at the same time, CR3 activation can exacerbate ICH-induced brain injury.

C1q/C3-CR3 signaling regulates phagocyte-mediated phagocytosis in different diseases. In stress-mediated visceral hypersensitivity, C1q, released from activated microglia, bounds target synapses, and triggered the cleavage of C3. The downstream cleavage product of C3 bounded to CR3 on the surface of microglia to modify synaptic plasticity and mediate visceral pain (15). In *E. coli*-mediated inflammatory responses, inhibition of phagocytosis and oxidative burst was obtained in monocytes when C3 activation was blocked. Additionally, anti-CR3 antibodies obviously reduced phagocytosis and oxidative burst (33, 34). Moreover, CR3 played an important role on NADPH oxidase activation and dopaminergic neurodegeneration in Parkinson’s disease (35). Both genetic ablation of CR3 and CR3 agonist LA-1 markedly alleviated oxidative stress in a mouse model of Parkinson’s disease (36). In animal models of murine and human cancer, pharmacological activation of CR3 with LA-1 promoted pro-inflammatory macrophage polarization (17). The role of C1q/C3-CR3 signaling is still unclear in ICH. Therefore, our research investigates the effect of CR3 after ICH and reveals that activation of CR3 with LA-1 significantly worsens ICH-induced neurological deficit with aggravated oxidative stress and neuronal cell death. Furthermore, we explored the effect of CR3 activation on microglia-mediated hematoma resolution after ICH. The Hp-CD163-HO-1 is the major pathway of microglia-mediated hematoma clearance (23, 37). Evidence showed that CD163/HO-1 upregulation after ICH was influenced by the complement system (8, 9). Treatment with N-acetyl-heparin (NAH), an inhibitor of complement cascade activation, significantly reduced perihematomal CD163-positive cells and HO-1-positive cells after ICH. Similarly, treatment with aurin tricarboxylic acid, an inhibitor of MAC formation, reduced CD163 and HO-1 expression after ICH. Furthermore, C3-deficient mice had fewer HO-1 positive cells around the hematoma after ICH. However, no direct evidence confirmed that specific complement components could enhance CD163/HO-1 expression. We found that the CR3 agonist LA-1 prominently promoted CD163/HO-1 expression and reduced residual hematoma volume after ICH. While hematoma clearance may theoretically improve neurological outcome, we hypothesize that oxidative stress induced by CR3 ultimately exacerbates brain injury after ICH. Inhibition of CR3-induced oxidative stress and enhancement of hematoma clearance mediated by CR3 might be an ideal therapeutic strategy for alleviating brain injury after ICH.

Minocycline is a semisynthetic tetracycline derivative with high permeability and can penetrate the blood–brain barrier, which acts as a neuroprotective agent and has a widely explored underlying mechanism (38). Minocycline has multiple mechanisms for treating ICH, including anti-inflammatory properties, anti-oxidant properties, MMP inhibition, and the alleviation of glutamate excitotoxicity (39). However, the effect of minocycline on the complement system is still small. In visceral pain, minocycline, which repressed microglial activation, suppressed transcription of C1q and CR3, and ultimately repressed C1q/C3-CR3-mediated synaptic engulfment in the amygdala, reduced visceral hypersensitivity (15). Furthermore, minocycline can decrease both the number of C3^+^/GFAP^+^ astrocytes and C3aR^+^/Iba-1^+^ microglia after germinal matrix hemorrhage (32). Consistently, we revealed that minocycline exerted neuroprotection, alleviated oxidative stress, and downregulated C1q and CR3 expression. However, the beneficial effects of minocycline were abolished by the CR3 agonist LA-1. We deduced minocycline alleviated ICH-induced brain injury *via* at least partial inhibition of CR3 expression. Then we further explored the effect of minocycline on hematoma resolution. We found less CD163-HO-1 expression and bigger hematoma volume in minocycline treatment as compared to the ICH control group. Based on our experiment result, we proposed minocycline reduced CD163-HO-1 level after ICH *via* partly repressing CR3 expression. Hematoma volume after ICH depends on hematoma resolution, including hematoma lysis and hematoma clearance (5). C1q, an initiating protein of the classical complement pathway, activated other complement components, and ultimately induced MAC-mediated erythrolysis. Based on the fact that minocycline reduced C1q expression and resulted in bigger hematoma volume after ICH, we inferred that neuroprotection of minocycline partly results from downregulation of C1q and ameliorating hematolysis. Furthermore, we explored the effects of oral minocycline treatment after ICH. Minocycline had two treatment routes, including intraperitoneal injection and oral gavage in animal experiments and, correspondingly, intravenous injection and oral administration in clinic treatment (40–43). Intraperitoneal injection is the main administration route in studies on ICH (24, 44, 45). In the randomized controlled trial of minocycline, patients with acute intracerebral hemorrhage received intravenous minocycline as treatment (46). Oral administration of minocycline after ICH lacks sufficient study. Long-term treatment with minocycline *via* oral gavage improved neurological function and inhibited neuroinflammation in Alzheimer’s disease (42). Our results confirmed that oral administration with minocycline exerted neuroprotection and alleviated neuronal death after ICH. Furthermore, oral administration is a more convenient treatment and has more value in clinical transformation than intravenous injection. Therefore, we proposed oral gavage as a potential route for minocycline treatment after ICH.

However, our research has some undeniable limitations. Firstly, we merely focused on a specific part of the complement pathway and other complement molecular areas were not further investigated. Second, both age and sex are significant factors in the complement system. In a cohort study of healthy Caucasian adults, it was demonstrated that women had significantly lower activity of alternative pathways in serum compared to men, whereas the elderly had obviously higher classical and alternative pathway activity in serum than the young (47). The effects of age and sex were not considered in our current study and deserve deeper exploration. Third, previous studies have shown that the neuroprotective effects of minocycline on ICH are mainly due to inhibition of microglia activation and a reduction of brain iron deposition. The effects of minocycline on microglial activation and brain iron deposition were not evaluated in this study. Fourth, the dose of oral minocycline administered in this study was based on previous studies of intraperitoneal administration. The dose–effect relationship of oral minocycline administration should be investigated in this study. Moreover, side effects of oral minocycline treatment, such as diarrhea, were not evaluated.

In conclusion, C1q/C3-CR3 signaling was activated with time after ICH. CR3 activation exacerbated ICH-induced brain injury, as well as enhanced CD163/HO-1 expression and promoted hematoma clearance. Oral minocycline administration improved neurological dysfunction and reduced hematoma resolution *via* partly inhibiting the C1q/C3-CR3 pathway. These results suggest that targeting C1q/C3-CR3 might be a novel and promising therapeutic strategy after ICH.

## Data availability statement

The datasets presented in this study can be found in online repositories. The name of the repository and accession number can be found below: NCBI Gene Expression Omnibus; GSE206971

## Ethics statement

This study was reviewed and approved by the Institutional Ethics Committee of the Second Affiliated Hospital, Zhejiang University of Medicine.

## Author contributions

SC and FL conceived and designed the study. YZ and LF performed the ICH model and immunofluorescence. SX and CX performed western blot and behavior test. QY and ZZ collected and analyzed clinic data. XF and HC provided and analyzed RNA sequence data. HZ, YP, and KY prepared the figure. SC and FL wrote the paper. All authors contributed to the article and approved the submitted version.

## Funding

This work was supported by the National Natural Science Foundation of China (grant numbers 81801144, 81971099, 81870908, 82171273, and 82171275) and the Key R&D Program of Zhejiang (2022C03133).

## Conflict of interest

The authors declare that the research was conducted in the absence of any commercial or financial relationships that could be construed as a potential conflict of interest.

## Publisher’s note

All claims expressed in this article are solely those of the authors and do not necessarily represent those of their affiliated organizations, or those of the publisher, the editors and the reviewers. Any product that may be evaluated in this article, or claim that may be made by its manufacturer, is not guaranteed or endorsed by the publisher.

## Supplementary material

Supplementary Figure 1 Experimental animal groups and mortality.

Supplementary Figure 2 Experimental design.

Supplementary Figure 3 Quantitative analyses of complement lectin pathway in transcriptive level in hematoma edge after ICH. n=3.
