# Supplementary figures and images for: Role of complement C1q/C3-CR3 signaling in brain injury after experimental intracerebral hemorrhage and the effect of minocycline treatment
Source: Front Immunol. 2022 Sep 15;13:919444. doi: 10.3389/fimmu.2022.919444 (PMC9520460; doi:10.3389/fimmu.2022.919444)

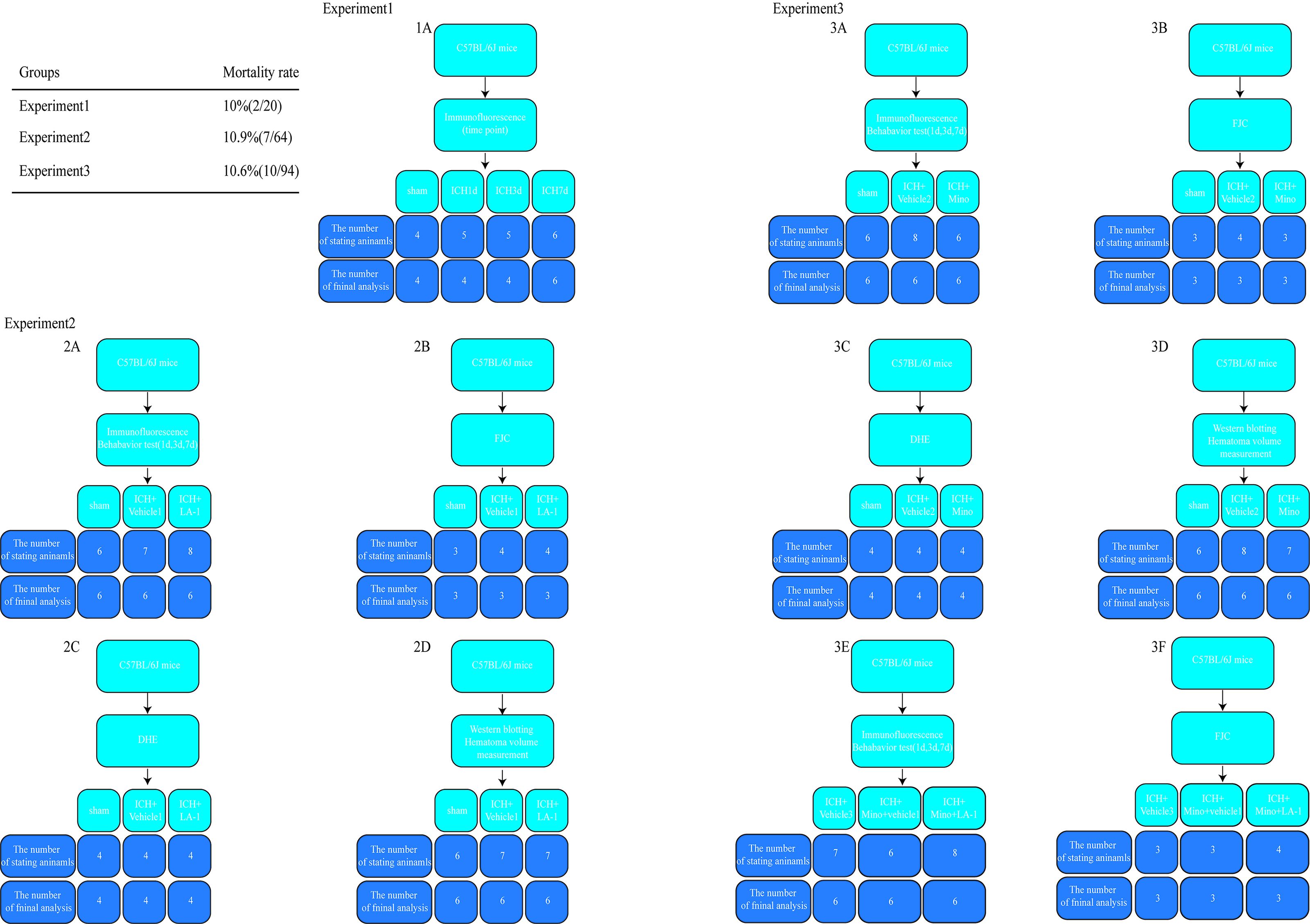

Supplement: Supplementary Figure 1 — Experimental animal groups and mortality. [file Image_1.tif]

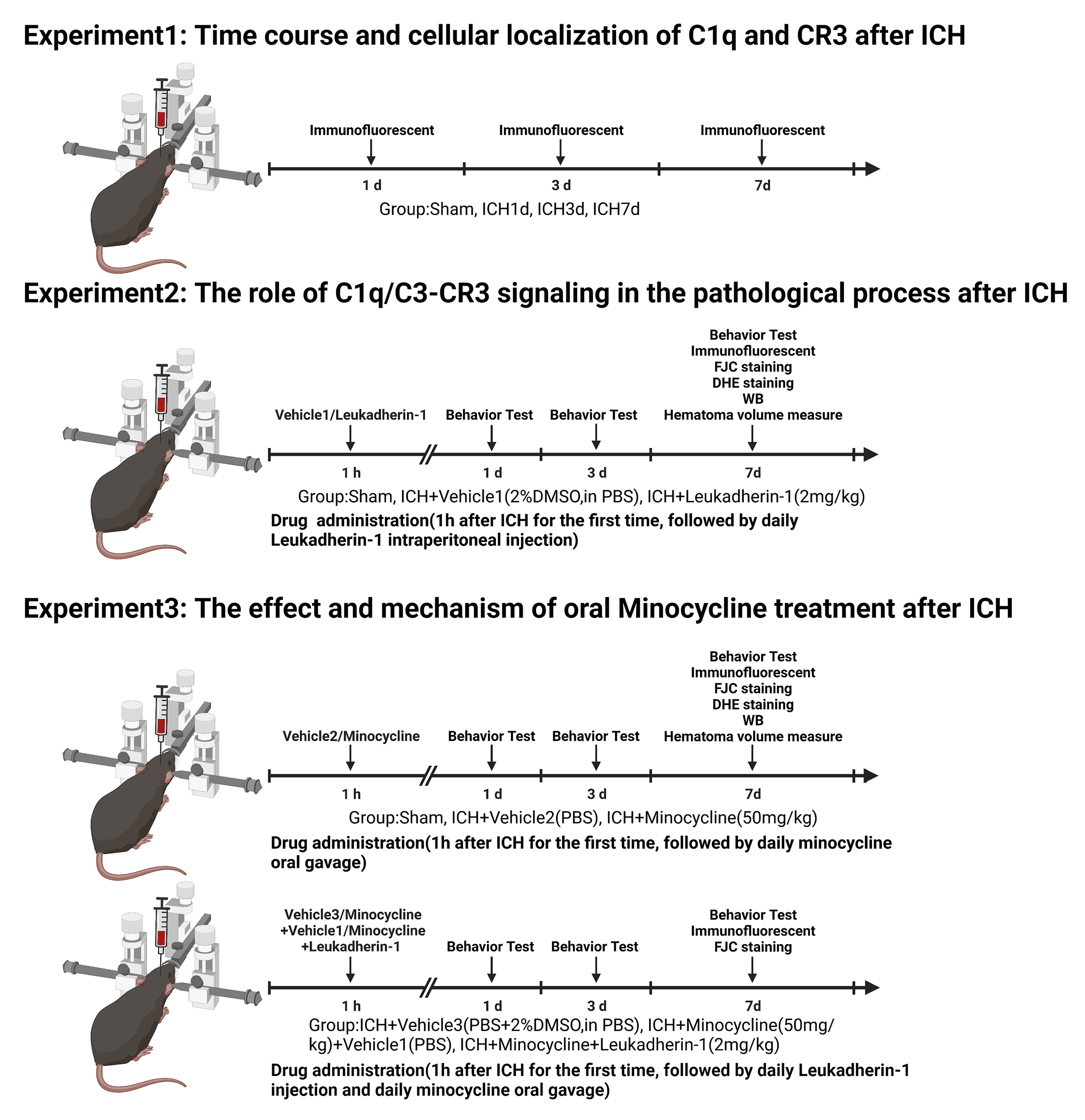

Supplement: Supplementary Figure 2 — Experimental design. [file Image_2.tif]

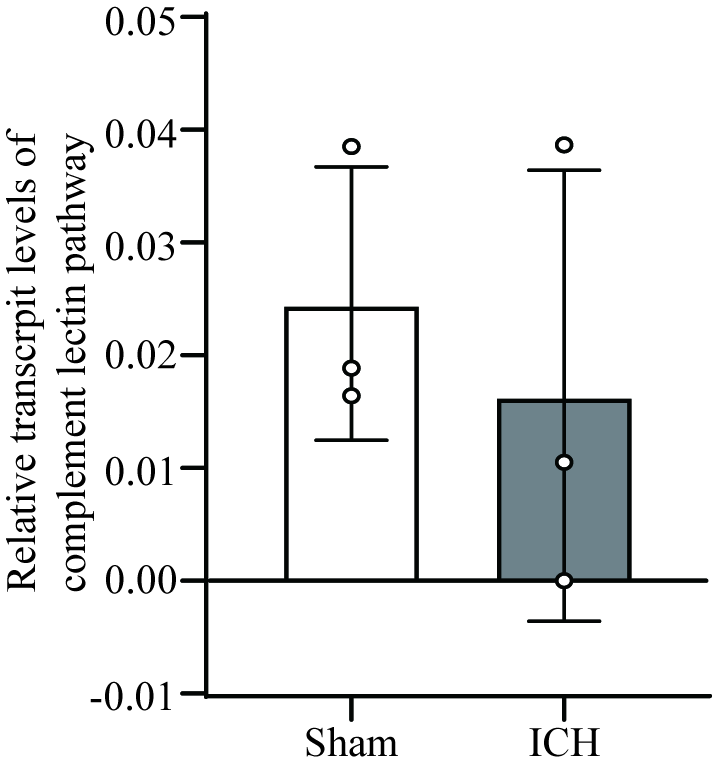

Supplement: Supplementary Figure 3 — Quantitative analyses of complement lectin pathway in transcriptive level in hematoma edge after ICH. n=3. [file Image_3.tif]
